# The sporulation of the green alga *Ulva prolifera* is controlled by changes in photosynthetic electron transport chain

**DOI:** 10.1038/srep24923

**Published:** 2016-04-22

**Authors:** Hui Wang, Apeng Lin, Wenhui Gu, Li Huan, Shan Gao, Guangce Wang

**Affiliations:** 1 Institute of Oceanology, Chinese Academy of Sciences, Qingdao 266071, China; 2 University of Chinese Academy of Sciences, Beijing 100049, China; 3 Nantong Branch, Institute of Oceanology, Chinese Academy of Sciences, Nantong 226006, China

## Abstract

Sporulation and spore release are essential phases of the life cycle in algae and land plants. *Ulva prolifera*, which is an ideal organism for studying sporulation and spore release, was used as the experimental material in the present study. The determination of photosynthetic parameters, combined with microscopic observation, treatment with photosynthetic inhibitors, limitation of carbon acquisition, and protein mass spectrometry, was employed in this experiment. Cycle electron transport (CEF) was found enhanced at the onset of sporangia formation. The inhibition effect of dibromothymoquinone (DBMIB) towards sporulation was always strong during the sporulation process whereas the inhibition effect of 3-(3′,4′-dichlorophenyl)-1,1-dimethylurea (DCMU) was continuously declined accompanied with the progress of sporulation. The changes of photosynthesis resulted from the limitation of CO_2_ acquisition could stimulate sporulation onset. Quantitative protein analysis showed that enzymes involved in carbon fixation, including RUBISCO and pyruvate orthophosphate dikinase, declined during sporogenesis, while proteins involved in sporulation, including tubulin and centrin, increased. These results suggest that enhanced cyclic electron flow (CEF) and oxidation of the plastoquinone pool are essential for sporangia formation onset, and changes in photosynthetic electron transport chain have significant impacts on sporulation of the green algae.

Sporogenesis is a developmental process that produces spores through cell division and differentiation. Reproduction via spores is an important and common reproductive method in land plants, algae, fungi, etc. and played a significant role in the exploration of land over the course of plant evolution^1^; additionally, sporulation is essential phases of the life cycle in cryptogams. During sporulation in land plants and algae, solar energy is absorbed and transferred by photosystems and eventually, together with carbon dioxide, is transformed into biomass through the carbon fixation cycle to support these energy-requiring processes. Photosynthesis not only provides energy and biomass for the plant growth but also regulates the involved physiological processes^2^. Photosynthesis consists of two portions, the light and dark reactions. These two portions can both influence the regulation of physiological processes in land plants and algae. Previous reports suggest that green alga *Chlamydomonas reinhardtii* undergoes state transition via sensing redox status of the plastoquinone pool^3^,^4^. Campbell *et al.*^5^ suggested that in cyanobacteria the redox status of the plastoquinone pool, which is a component of the photosynthetic electron transport chain, can regulate cellular differentiation, including the formation of hormogonia for reproduction^5^. While Mine *et al.*^6^ and Yamagishi *et al.*^7^ determined that many environmental factors affecting photosynthesis, such as the concentration of dissolved inorganic carbon and light quality, can influence the release of gametes or the formation of zoospores in green alga *Bryopsis plumose*^6^,^7^. Pearson *et al.*^8^ also confirmed this viewpoint in fucoid algae^8^,^9^. Although these algae are rather distinct with each other^10^, regulation mechanism in the formation of reproductive structure (hormogonia or gametes) is similar in many aspects. Therefore, it is feasible that photosynthetic electron transport chain may regulate spore (gamete) formation and release in all photosynthetic organisms. However, we still know little about the changes of photosynthesis during sporulation and how these photosynthetic changes can influence sporulation process in photosynthetic organisms.

*Ulva prolifera* (O.P. Muller) J. Agardh (Ulvales, Chlorophyta) is widely distributed in coastal waters. It is one of the most common fouling macroalgae that cause green tide and the protagonist of world’s largest macroalgal bloom along Qingdao in June 2008^11^,^12^. The species is a member of the green algae which are relative with land plants in phylogeny and share the same pigments^13^. Additionally, *U. prolifera* can be easily cultured in the laboratory. The life history of the species includes morphologically similar haploid and diploid phases, both of which can reproduce by haploid or diploid asexual zoospores derived from vegetative cells^14^; moreover, *U. prolifera* can also reproduce sexually by anisogametes^15^. When the thallus of *U. prolifera* is cut into small disks, the vegetative cells often rapidly develop into sporangia and release spores^16^. Hence, *U. prolifera* is an ideal organism for studying spore (gamete) formation and spore (gamete) release in photosynthetic organisms.

In this study, we employed *U. prolifera* as an experimental material to investigate the changes of photosynthesis, especially concerning photosynthetic electron transport chain, during sporulation and spore release. We found that photosynthesis indeed experiences significant changes during sporulation. If photosynthesis is under the influence of external factors that can change photosynthesis, sporulation is consequently greatly affected. Sporulation and spore release can be controlled by adjusting photosynthesis. These results suggest that photosynthesis plays a central role in the regulation of spore formation and release in photosynthetic organisms including green algae.

## Results

### Sporulation and changes of photosynthetic parameters

**Figure 1:**
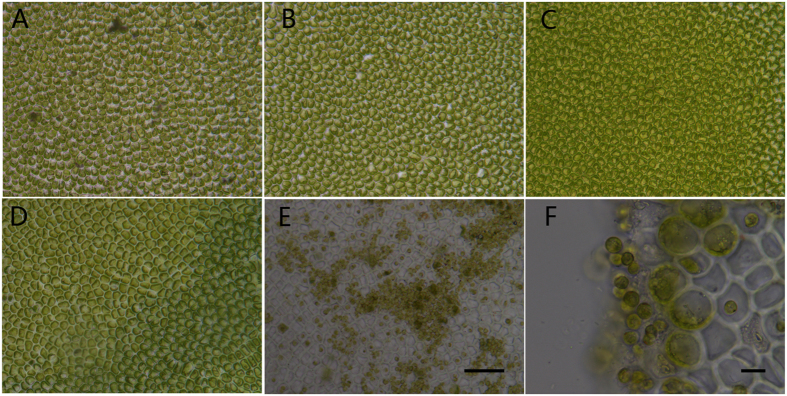
Cells of *Ulva prolifera* in excised disks from 0 to 60 h in culture. (**A**) Cells in newly excised disks at 0 h; (**B**) cells at 24 h; (**C**) cells at 36 h; (**D**) cells at 48 h; (**E**) the completion of sporangia formation and spore release at 60 h; (**F**) spores at 60 h. All cells in (**A**–**D**) are vegetative. When numerous spores were released from disk, the formerly green disk turned into transparent disk because remains are transparent white cell wall. The scale bar represents 50 μm in (**A**–**E)**. The scale bar represents 10 μm in (**F**).

Under our regular microscopic observation of the excised disks in culture through sporulation and spore with four flagella release, the formation of sporangia did not occur before 48 h (Fig. 1A–D), but sporulation and spore release occurred by 60 h (Fig. 1E,F). Therefore, the formation of sporangium must have occurred from 48 to 60 h.

**Figure 2:**
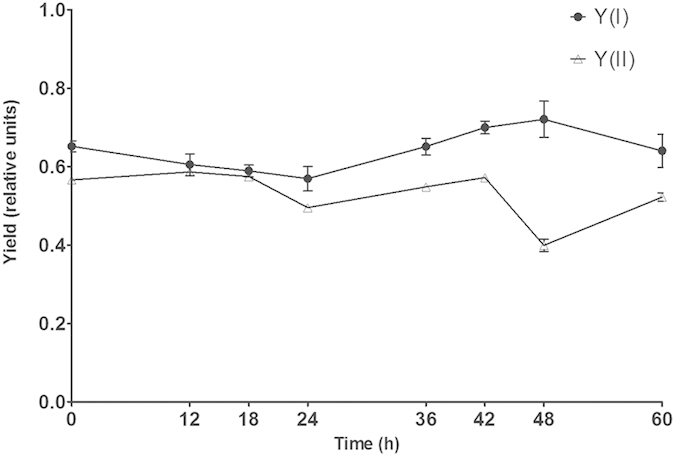
The changes of Y(I) and Y(II) from excised disks through sporulation and spore release from 0 to 60 h. Each parameter was determined at least three times. Error bars indicate the standard deviation.

Numerous photosynthetic parameters, including effective PS II quantum yield (Y(II)) and photochemical quantum yield of PS I (Y(I)), were measured and recorded during the culture of the excised disks through sporulation and spore release, and the trends of these photosynthetic parameters were obtained (Fig. 2). We found that Y(II) obviously decreased, while Y(I) increased, at 48 h in culture. From 0 to 48 h in culture, Y(I) and (especially) Y(II) were relatively stable, and the variation tendencies between them were similar. From 48 to 60 h, the changes of Y(II) and Y(I) occurred, and more importantly, the variation tendencies between the two parameters were opposite. At 48 h, Y(II) dropped to its lowest level, while Y(I) rose to its highest level. In contrast, Y(II) ascended sharply as Y(I) declined at 60 h.

### Inhibitions of sporulation and spore release by the addition of photosynthetic inhibitors

**Figure 3:**
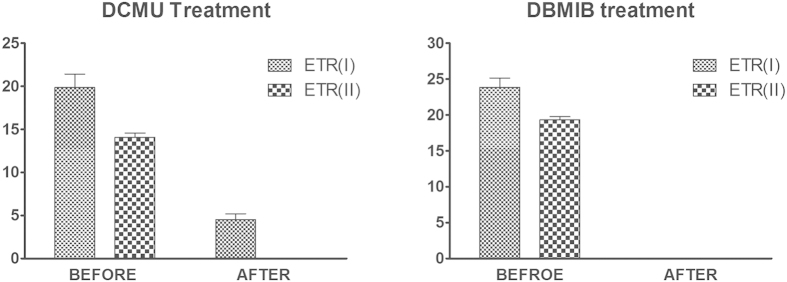
The inhibition effect of DCMU and DBMIB. DCUM treatment graph shows that after 10 μM DCMU treatment, the electron transport rate of PS II (ETR(II)) declined to zero and the photosynthetic electron transport rates of PS I (ETR(I)) remained low value. This result means that PS II was completely inhibited by DCMU and remaining ETR(I) may come from cyclic electron flow in PS I. DBMIB treatment graph shows that after 10 μM DBMIB treatment, both ETR(I) and ETR(II) declined to zero. This result means that PS II and PS I was completely inhibited by DBMIB meanwhile cyclic electron flow was also cut off.

The photosynthetic inhibitors DCMU and DBMIB were applied in the DCMU and DBMIB treatment groups, respectively. Both inhibitors were confirmed to be effective (Fig. 3). Sporulation and spore release were not observed in the DBMIB or DBMIB + ascorbate treatment groups (Table 1). There was no sign of sporangia in any DBMIB or DBMIB + ascorbate group. DBMIB appeared block the process of sporangium formation. Similarly, sporulation and spore release were not observed in the DCMU group 1 (The addition at 0 h). In contrast, sporulation and spore release occurred in DCMU groups 2, 3, and 4 (The addition at 24 h, 36 h, or 48 h) (Table 2). There were differences concerning sporulation and spore release between these three groups. In DCMU group 2 it was observed that the longest time was required for complete sporangium formation and spore release. While DCMU group 3 spent the second longest time. We found the formation of sporangium partially finished in DCMU group 2 and 3. Sporangium formation and spore release occurred at the same time in group 4 as in the control group. In addition, the mobility of spores was unaffected by the addition of DCMU. All statistical data are shown in the Table 1 and Table 2.

### Sporulation and spore release in correlation with dissolved inorganic carbon (DIC) (NaHCO_3_) concentrations in culture

In both experiments concerning different DIC concentrations, we counted the sum of the transparent disks and the sum of the green disks with the naked eye using the background contrast method. As revealed by microscopic observation, the transparent disks had released numerous spores, while the green disks that contain numerous vegetative cells or (and) sporangium had not yet released spores on a large scale. The proportion of spore release was calculated as the sum of transparent disks divided by the total sum of disks in the group. In the first experiment, the proportions of spore release were 39.3%, 30.8%, and 25.9% (all values represent means, n = 3) in DIC groups 1, 2, and 3, respectively (Table 3).

In the second experiment, the proportions of spore release were 2.2%, 78.2%, and 20.8% (all values represent means, n = 3) in DIC groups 4, 5, and 6, respectively (Table 4). Almost no sporulation occurred in the disks of group 4. In contrast, despite the release of few spores, many sporangia were formed in group 6.

### Contrast of photosynthetic parameters in correlation with different dissolved inorganic carbon (NaHCO_3_) concentrations in culture

**Figure 4:**
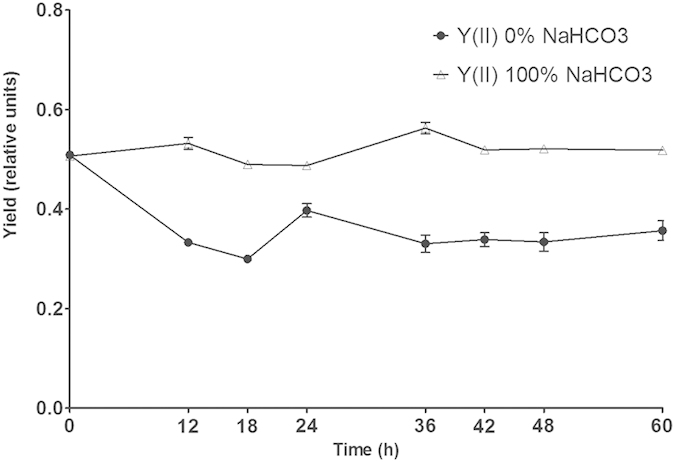
Contrast of Y(II) between 0% and 100% NaHCO_3_ Concentration in culture from 0 to 60 h. Each parameter was determined at least three times. Error bars indicate the standard deviation.

Under concentrations of 0% and 100% NaHCO_3_ cultures respectively, the contrasts between photosynthetic parameters especially Y(II) were apparent. Under concentrations of 100% NaHCO_3_ culture, Y(II) stayed relative stable and high value from 0 to 60 h. Contrast with concentrations of 100% NaHCO_3_ culture, Y(II) declined sharply from 0 to 18 h and kept a relative low value in concentrations of 0% NaHCO_3_ culture (Fig. 4).

### The analysis of proteins at different culture times

**Figure 5:**
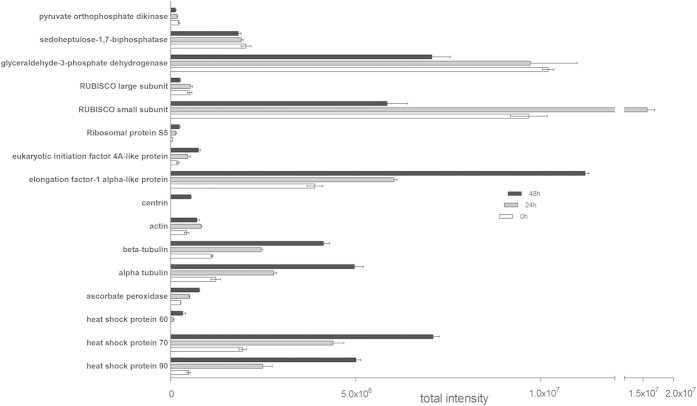
Contrast of protein abundances at 0, 24, and 48 h in culture. Error bars indicate the standard deviation.

All disks whose proteins were extracted and analyzed were cut from same sample and cultured under the same conditions. The only difference between the three groups was culture time. The analysis of protein differences between the three groups was conducted through the contrast analysis of proteins at 0, 24, and 48 h. As shown in Fig. 5, the protein abundances were obviously distinguishable between the 0, 24, and 48 h groups. The abundances of stress proteins, such as heat shock protein 90, at 48 h were 10-fold higher than those observed at 0 h. The rapid accumulation of stress proteins was accompanied by the increase of culture time. Similarly, the abundance of cytoskeletal proteins such as tubulin and actin increased from 0 to 48 h. Centrin, a protein component of centrosomes and mitotic spindle poles^17^, was not found at 0 or 24 h but was detected at 48 h. Translation proteins increased to various degrees from 0 to 48 h. Most of the enzymes in the Calvin cycle also exhibited a downward trend from 0 to 48 h. The abundance of the RUBISCO small subunit reached its maximum at 24 h and then decreased at 48 h. The peptide sequences of the protein contents in Fig. 5 can be found as Supplementary Table S1.

## Discussion

Although both DCMU and DBMIB are photosynthetic inhibitors, which can lead to great changes in the photosynthetic electron transport chain, it is interesting that there are evident differences in inhibition effect towards sporulation between DCMU and DBMIB. Our results indicate that the inhibition effect of DCMU is to inhibit completely (The addition at 0 h), to slow down the sporulation process (The addition at 24 h or 36 h), or ineffective (The addition at 48 h); while DBMIB can block completely the sporulation process anytime. DCMU is known as an inhibitor of photosystem II and inhibits linear electron flow (LEF)^18^. Our photosynthetic parameters observations show that prominent LEF and moderate cyclic electron flow (CEF) occurred from 0 to 48 h. The cooperation of LEF and CEF may help the Calvin cycle reach its optimal performance to support sporulation^2^,^19^. In DIC group 4 (completely null DIC group) the complete inhibition towards sporulation was caused by disability in the Calvin cycle, which the lack of available carbon leads to. The mechanism of DCMU in sporulation inhibition is to block LEF and then constrain the Calvin cycle. The results in DIC group 4 (completely null DIC group) and DCMU group 1 (The addition at 0 h) are consistent with the results of Yamagishi *et al.*^7^ that nuclei did not divide in darkness or in the presence of 1 μM DCMU^7^. Therefore available Calvin cycle is essential to the sporulation process. But for sporulation the demand to Calvin cycle declined continuously from 0 h to 48 h. In DIC 1 and 5 the lack of available carbon since 24 h or 36 h did not inhibit the sporulation completely. Our analysis of proteins also shows most of the enzymes in the Calvin cycle, especially RUBISCO, exhibited a downward trend over this period. Consequently the inhibition effect that is correlated with the Calvin cycle is alleviated accompanied with the process of sporulation. This is why the inhibition capacity of DCMU continuously declined to be invalid from 0 h to 48 h during sporulation. The result that the addition of DCMU at 48 h is ineffective to inhibit suggests that the all sporulation process depend on the Calvin cycle has been finished at 48 h.

However DBMIB also can cut off LEF to constraint the Calvin cycle, this inhibition mechanism can not explain why there is evident inhibition contrast between DCMU and DBMIB addition at 48 h. According to photosynthetic parameters, at 48 h previous coordination of LEF and CEF for the Calvin cycle had been broken while CEF was enhanced markedly. CEF can induce the acidification of the thylakoid lumen and enhance the ATP supply for mitosis in sporangium formation and energy stocking for spore swimming^20^. We reckon that CEF may be essential for normal sporulation process. DBMIB can cut off CEF to inhibit sporulation by hampering ATP support while DCMU allows the running of CEF^18^,^21^. But blocking CEF is just secondary section of DBMIB inhibition mechanism. It still can not explain complete inhibition caused by DBMIB because ATP short supply can be supplemented by other metabolic pathways. The result that the addition of DCMU at 48 h is ineffective to inhibit demonstrates that all changes in the photosynthetic electron transport chain caused by DCMU at 48 h have no impact towards the sporulation process. DCMU can not only block LEF but also maintain an oxidized plastoquinone pool, which is an important component in photosynthetic electron transport chain^21^. It means that the onset of sporangium formation and spore release, which happened after 48 h, can be carried on without any inhibition even though plastoquinone pool keeps oxidized. On the contrary, DBMIB can keep the plastoquinone pool reduced. Moreover, the imbalance between Y(I) and Y(II) at 48 h can also make plastoquinone pool more relatively oxidized (Fig. 2). It is known that redox state of the plastoquinone pool play a significant role in signal transduction and gene expression^22^,^23^. In cyanobacteria, an oxidized plastoquinone pool can initiate the differentiation of hormogonia, a sort of reproductive structure^5^. We propose that the oxidized state of plastoquinone pool is essential to initiating sporangium formation. The oxidized state may severed as preliminary signal to activate subsequent cascade process concerning sporulation especially sporangium formation. Permanent reduced state of plastoquinone pool created by DBMIB eliminates this preliminary signal so that DBMIB completely inhibit sporulation anytime. Higher proportion of spore release in DIC group 1 and group 5 than DIC group 2 and group 6 also is correlated with the oxidized state of plastoquinone pool and CEF. The stress of limited available carbon acquisition in DIC group 1 and group 5 leads to not only downregulation of LEF, which showed in Fig. 4, but also the enhancement of CEF^24^. More importantly, this stress results in the oxidized state of plastoquinone pool^25^. The oxidized state of plastoquinone pool combined with enhanced CEF accelerates sporangium formation onset and spore release when some cells have finished sporulation processes that are dependent on available carbon. This view can also explain well why low dissolved carbon (NaHCO_3_) concentrations caused by low water motion can stimulate spore release of fuciod algae in natural environment^8^.

In conclusion, our results confirmed that the photosynthetic electron transport chain participates in regulation of the entire sporulation process including early preparations concerning the Calvin cycle and sporangium formation. Enhancement of CEF and the oxidized state of plastoquinone pool are essential to normal sporangium formation.

**Figure 6:**
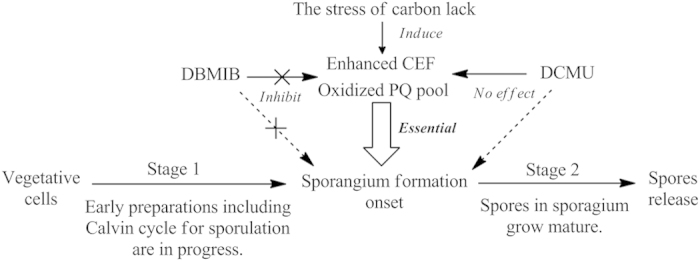
Photosynthetic regulation of sporulation and spore release in experiments.

According to our study especially quantitative protein analysis integrated with the relevant results, the entire sporulation process should be divided into two stages. Stage 1 lasted from 0 to 48 h. During this stage all cells were vegetative and made early preparations for sporulation including the Calvin cycle operation. This stage is similar to first 2 days in main culture of *Bryopsis plumosa* that needs the supplement of NaHCO_3_ described by Yamagishi *et al.*^7^. The abundances of proteins those are required to sporulation such as tubulin and translation proteins increased continuously over this period. Tubulin is essential for algal zoospore flagella and the mitotic spindle apparatus^26^,^27^. In addition, stress proteins, especially heat shock proteins, rapidly accumulated because some heat shock proteins play important roles in sporulation among eukaryotic cells^28^. Stage 2 began at 48 h. The onset of sporangium formation was initiated by enhancement of CEF and the oxidized state of plastoquinone pool in this stage. Centrin, which is essential to centrosome duplication during mitosis and in algal microtubule-organizing centers, was found at 48 h^29^. Finally spores grew mature and released at later period in Stage 2. Figure 6 outlines our current understanding concerning how changes in photosynthetic electron transport chain influence the sporulation process.

## Materials and Methods

### Experimental materials and culture conditions

*U. prolifera* were collected carefully from the coast of Qingdao (06/08/2014). The thalli were washed in sterile seawater to remove any attachment. To accelerate the sporulation of *U. prolifera* and obtain a high degree of synchronous spore formation^16^,^30^, the thallus was cut into small disks using a Stiletto apparatus (Harris Uni-core, USA). These disks were 0.5, 0.75, 1.0, and 1.2 mm in diameter. We determined that the 0.75 mm disks were the most appropriate size because they developed sporangia in 48–60 h and were easy to handle. Therefore, the 0.75 mm disks were employed in the subsequent experiments. The disks were cultured in common sterile seawater (salinity 30 psu) at 21 °C with 30–35 μmol photon m^−2^s^−1^ (Light: Dark = 12 h: 12 h). All experiments were under same illumination rhythmicity.

### Microscopic observations and determinations of photosynthetic parameters from excised disks during sporulation and spore release

The cells of the 0.75 mm disks were regularly observed under the microscope from cutting through spore release. Photographs of the developmental processes, including sporangia formation and spore release, were recorded at regular time intervals using a differential interference contrast microscope (Leica DM2500, Germany). The determinations of photosynthetic parameters were conducted at the same time as the microscopic observations using a chlorophyll fluorometer (Dual-PAM-100 Measuring System, Waltz GmbH, Effeltrich, Germany). The time points of the microscopic observations and determination of photosynthetic parameters in the culture were 0, 12, 18, 24, 36, 42, 48, and 60 h. A sufficient number of disks were set on a thin microscopic glass slide and received 5 minutes of dark adaption before measurement. Light emitted by a 620 nm light-emitting diode (LED) and blue actinic light at 100 μmol photons m^−2^s^−1^ emitted by 460 nm LED arrays were delivered to these disks for 5 min periods by a DUAL-DR measuring head. Additionally, saturating light pulses of 300 ms duration and 10,000 μmol photons m^−2^s^−1^ were given. The effective photosystem II (PS II) quantum yield (Y(II))^31^ and photochemical quantum yield of photosystem I (PS I) (Y(I))^32^ were automatically calculated by Dual-PAM software. According to Genty *et al.*^33^, the effective PS II quantum yield (Y(II)) was calculated by equation (1)^33^:


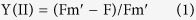


The photochemical quantum yield of PS I (Y(I)) was calculated by equation (2):


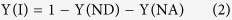


where Y(ND) represents the fraction of overall P700 that is oxidized in a given state, and Y(NA) represents the fraction of overall P700 that cannot be oxidized by a Saturation Pulse in a given state due to a lack of acceptors.

### Photosynthetic inhibitor treatments

To study how photosynthetic electron transport influences sporulation and spore release, the photosynthetic inhibitors 3-(3′,4′-dichlorophenyl)-1,1-dimethylurea (DCMU) (Sigma Aldrich) and dibromothymoquinone (DBMIB) (Sigma Aldrich) were used to treat the 0.75 mm disks cultured in common sterile seawater respectively. The control was the normal group without DCMU or DBMIB. The final concentrations of DCMU and DBMIB were 10 μM. In order to prevent DBMIB to act as a PS II electron acceptor, we used 1 mM ascorbate to keep DBMIB reduced^34^. The DCMU treatments were conducted in sets of disks designated as groups 1, 2, 3, and 4, which had been previously cultured for 0, 24, 36, and 48 h, respectively. After treatment with the inhibitor, these groups continued to be cultured until reaching a total of 84 h. The DBMIB treatments followed the same protocol. The status of these groups was regularly monitored and recorded by microscope (Leica DM2500, Germany).

### Disks cultures under different dissolved inorganic carbon (DIC) (NaHCO_3_) concentrations

In this study, artificial seawater (NaCl 20.758 g, KBr 0.0845 g, Na_2_SO_4_ 3.477 g, boric acid 0.0225 g, KCl 0.587 g, NaF 0.0027 g, MgCl_2_•6H_2_O 9.395 g, CaCl_2_•2 H_2_O 1.316 g, SrCl_2_•6H_2_O 0.0214 g per liter) was used to culture the 0.75 mm disks, as the concentration of dissolved inorganic carbon (DIC) (NaHCO_3_) in artificial seawater can be adjusted. Concentrations of 0%, 20%, and 100% NaHCO_3_ were employed for the different DIC concentration groups. In artificial seawater, a 100% concentration of NaHCO_3_ is achieved at 2 mM. To prevent outside carbon sources, especially atmospheric CO_2_, from interfering with the results, a homemade apparatus was employed to supply airflow without CO_2_ while keeping the culture protected from the surrounding air. In the experiments, a 100% concentration of NaHCO_3_ was used for the first 24 h of artificial seawater culture, and 0%, 20%, and 100% concentrations of NaHCO_3_ were then employed from 24 to 60 h in DIC groups 1, 2, and 3, respectively.

In another set of experiments, 0%, 100%, and 100% concentrations of NaHCO_3_ in artificial seawater were used for the first 36 h of culture, followed by concentrations of 0%, 0%, and 100% from 36 to 60 h, in DIC groups 4, 5, and 6, respectively.

### Intact thalli cultures under different dissolved inorganic carbon (DIC) (NaHCO_3_) concentrations and determinations of photosynthetic parameters

Concentrations of 0% and 100% NaHCO_3_ were employed for culturing intact thalli respectively. Culture conditions were consistent with disks cultures under different DIC concentrations described above. The time points of determination of photosynthetic parameters in the culture were 0, 12, 18, 24, 36, 42, 48, and 60 h.

### Total soluble protein extraction and quantitative protein analysis

Total soluble protein was extracted from 0.75 mm disks cultured for 0, 24 and 48 h using the phenol method as Wang *et al.*^35^ described^35^. Protein concentration was determined using the Bradford method^36^. Protein solubilized in 8 M urea was reduced with 10 mM DTT at 37 °C for 1 h and alkylated with 50 mM iodoacetamide in the dark at 25 °C for 1 h. Sequencing-grade modified trypsin was used for in-solution digestion with trypsin: protein at 1:30 (w/w).

The tryptic peptides were then subjected to liquid chromatography–mass spectrometry (LC-MS/MS) for quantitative analysis. The peptides were loaded onto a 2.1 mm × 150 mm reverse-phase column (Zorbax SB-C18, Agilent) and eluted with gradient mobile phase solution into an Agilent 6520b Q-TOF mass spectrometer^37^. Data acquisition was performed in the auto MS-MS mode of MassHunter software (version B03.01, Agilent). The spectra were processed using SpectrumMill MS Proteomics Workbench (version A.03.03, Agilent) and searched against the NCBI green algae protein database (retrieved on April 21, 2013). The precursor mass tolerance was set at ± 20 ppm. All protein identifications with protein score ≥20 and peptide SPI ≥60% were considered as positive results. Finally, gene ontology (GO) analysis was conducted for protein function analysis.

## Additional Information

**How to cite this article**: Wang, H. *et al.* The sporulation of the green alga *Ulva prolifera* is controlled by changes in photosynthetic electron transport chain. *Sci. Rep.* **6**, 24923; doi: 10.1038/srep24923 (2016).

## Supplementary Material

Supplementary Information

## Figures and Tables

**Table 1:** Sporulation differences between groups with the addition of DBMIB or DCMU at 48 h.

Differences between groups with the addition of DBMIB or DCMU at 48 h	DCMU	DBMIB	DBMIB + ascorbate	Without DCMU or DBMIB
The proportion of sporulation at 60 h (mean)	55%	4%	3%	62%

We can find that compared with the normal group, the addition of DCMU at 48 h seldom inhibited the sporulation but the addition of DBMIB at 48 h significantly inhibited the sporulation.

**Table 2:** The proportion of sporulation in different DCMU treatment groups at 60 h.

Different DCMU treatment groups at 60 h	DCMU Group 1 (The addition at 0 h)	DCMU Group 2 (The addition at 24 h)	DCMU Group 3 (The addition at 36 h)	DCMU Group 4 (The addition at 48 h)
The proportion of sporulation at 60 h (mean)	4 ± 3%	28 ± 5%	51 ± 7%	67 ± 4%

We can find when DCMU was added at early stage of culture (0 h and 24 h), the inhibition effect towards sporulation is significant. When DCMU was added at late stage of culture (36 h and 48 h), the inhibition effect towards sporulation is less effective.

**Table 3:** Proportions of spore release in the first DIC experiment.

1^st^ DIC experiments	*Group*1(0 h–24 h 100%NaHCO_3_ → 24 h–60 h 0%NaHCO_3_)	*Group*2(0 h–24 h 100%NaHCO_3_ → 24 h–60 h 20%NaHCO_3_)	*Group*3(0 h–24 h 100%NaHCO_3_ → 24 h–60 h 100%NaHCO_3_)
The proportion of spore release (%)	38.9	30.1	26.6
	36.7	33.3	27.7
	42.4	29.1	23.3
Mean (%)	39.3	30.8	25.9

The proportions of spore release in the different groups are significantly different (p = 0.0126 < 0.05, by repeated measures ANOVA).

**Table 4:** Proportions of spore release in the second DIC experiment.

2^nd^ DIC experiments	*Group*4(0 h–36 h 0%NaHCO_3_ → 36 h–60 h 0%NaHCO_3_)	*Group*5(0 h–36 h 100%NaHCO_3_ → 36 h–60 h 0%NaHCO_3_)	*Group*6(0 h–36 h 100%NaHCO_3_ → 36 h–60 h 100%NaHCO_3_)
The proportion of spore release (%)	0	85.7	18.8
	6.7	84.6	23.5
	0	64.3	20.0
Mean (%)	2.2	78.2	20.8

The proportions of spore release in the different groups are significantly different (p = 0.0003 < 0.05, by repeated measures ANOVA).
