# Supplementary material for: The sporulation of the green alga Ulva prolifera is controlled by changes in photosynthetic electron transport chain
Source: Sci Rep. 2016 Apr 22;6:24923. doi: 10.1038/srep24923 (PMC4840353; doi:10.1038/srep24923)
Supplement: Supplementary Information [file srep24923-s1.pdf]

The sporulation of the green alga *Ulva prolifera* is controlled by changes in photosynthetic electron transport chain

Hui Wang[1,2,]*, Apeng Lin[1,]*, Wenhui Gu[1,3]*, Li Huan[1], Shan Gao[1], Guangce Wang[1,]†

[1] Institute of Oceanology, Chinese Academy of Sciences, Qingdao 266071, China

[2] University of Chinese Academy of Sciences, Beijing 100049, China

[3] Nantong Branch, Institute of Oceanology, Chinese Academy of Sciences, Nantong 226006, China

* These authors contributed equally to this work.

† To whom correspondence should be addressed. Tel: 86-532-82898574. E-mail: gcwang@qdio.ac.cn

Supplementary Table S1

The peptide sequences of the protein contents in Figure 5

heat shock protein 90	Sequence	SPI (%)	Spectrum intensity	MH+ Matched (Da)
	(R)VFIMDNCEELVPEWLSFVK(G)	89.2	1.77E+05	2355.14
	(K)GIVDSEDLPLNISR(E)	92	3.64E+05	1527.801
	(K)SDLINNLGTIAR(S)	92.7	3.82E+05	1286.706
	(K)SGDEATSLKDYVTR(M)	90.4	2.56E+05	1541.744
	(K)GLEVLFMVDPIDEYAVQQLK(E)	89.6	1.93E+05	2307.194
	(K)TLTIVDSGIGMTK(S)	92.5	4.02E+05	1335.719
	(K)GLEVLFMVDPIDEYAVQQLK(E)	83.9	8.65E+04	2307.194
	(R)VFIMDNCEELVPEWLSFVK(G)	82.8	2.02E+05	2355.14
	(R)ELISNSSDALDKIR(F)	61.1	1.69E+05	1560.823
	(K)VIYYITGESR(K)	85.7	2.80E+05	1200.626
	(K)LAELLR(F)	91.1	3.56E+05	714.451
	(R)NPDEVTKEEYGAFYK(S)	66.9	2.14E+05	1789.828
	(K)EDQLEYLEER(R)	86.8	1.27E+05	1323.606

	(R)ELISNSSDALDK(I)	83.5	1.69E+05	1291.638
	(K)MILFLK(E)	86.5	2.06E+05	764.474
	(K)SVLFVPK(R)	75.4	2.88E+05	789.487
	(K)DILADKVEK(V)	80.5	1.12E+05	1030.578
	(K)MEELD(-)	88.4	4.66E+04	636.255
	(K)FYEAFGK(N)	71.5	1.90E+05	861.414
	(R)FQSLTDK(S)	70.9	2.10E+05	838.431
	(K)FEELTR(V)	71.7	6.83E+04	794.404
	(R)EWDLLNK(Q)	66.5	2.20E+05	917.473
	(K)RAPFDMFDQR(K)	79.8	1.62E+05	1282.6
	(K)DILADKVEK(V)	68.9	5.06E+04	1030.578
	(K)EIFLR(E)	67.6	1.49E+05	677.398
	(K)SVLFVPK(R)	84.9	6.81E+04	789.487
	(K)ITEIK(D)	81.2	5.67E+04	603.371
	(R)DNSMSSYMSSK(K)	64.4	7.86E+04	1236.487

heat shock protein 70	Sequence	SPI (%)	Spectrum intensity	MH+ Matched (Da)
	(R)ITPSYVAFTDSER(L)	99	1.65E+05	1485.722
	(R)TTPSYVGFTDTER(L)	87.6	2.06E+05	1473.686
	(R)IINEPTAAAIAYGLDKK(S)	94.4	1.07E+06	1787.99

(R)IINEPTAAAIAYGLDK(K)	96	1.52E+05	1659.895
(R)FEELCMDLFR(K)	87.4	1.20E+05	1359.607
(K)VQSLLQEFFGGK(E)	85.4	4.31E+05	1352.721
(R)ARFEELCMDLFR(K)	89.7	2.25E+05	1586.745
(R)VEIISNDQGNR(T)	87.2	1.31E+05	1244.623
(K)SINPDEAVAYGAAVQAAILTGEGSSK(V)	88.6	2.46E+05	2519.262
(K)AVEETISWLDANQLAEVEEFESK(Q)	72	4.74E+04	2637.257
(K)DAGSIAGLEVLR(I)	94.3	3.73E+05	1200.658
(K)AAGDKPMLQVQYK(G)	76.1	3.08E+05	1448.757
(K)CMDPVEACMR(D)	72.5	1.08E+05	1268.489
(K)NQVAMNPTNTVFDAK(R)	84.4	2.14E+05	1649.795
(K)AVEETISWLDANQLAEVEEFESK(Q)	74.4	1.81E+05	2637.257
(K)VQDLLLLDVAPLSLGIETAGGVMTALIPR(N)	70.3	1.26E+05	2975.685
(K)NSLENYAYSMR(N)	93.1	1.34E+05	1347.6
(K)VQDLLLLDVAPLSLGI	75.6	1.99E+05	2975.685

ETAGGVMTALIPR(N)			
(K)ITITNDK(G)	85.5	2.09E+05	804.446
(R)ARFEELCMDLFR(K)	79.1	2.34E+05	1586.745
(R)VEIIANDQGNR(I)	89.1	1.14E+05	1228.628
(K)NAVVTVPAYFNDAQR(Q)	88.1	1.24E+05	1664.839
(K)QAVVTVPAYFNDGQR(Q)	77	1.67E+05	1664.839
(K)ELEGVCNPIISK(M)	84.1	1.36E+05	1358.698
(R)LIGDAAK(N)	79	1.69E+05	687.404
(K)QAVVTVPAYFNDGQR(Q)	70.6	2.32E+05	1664.839
(R)TVYDVK(R)	89.4	5.55E+04	724.388
(K)VQELLK(E)	84.4	1.31E+05	729.451
(K)VQDLLLLDVAPLSLGIETAGGVMTALIPR(N)	64.3	6.90E+04	2975.685
(K)DAGTIAGLNVAR(I)	74	1.52E+05	1157.627
(K)AVEETISWLDANQLAEVEEFESK(Q)	65.9	1.81E+05	2637.257
(K)FNDMAVQDDIK(H)	76.5	5.35E+04	1295.594
(K)VQDLLLLDVAPLSLGIETAGGVMTALIPR(N)	65.8	1.03E+05	2975.685

	(K)QAVVTVPAYFNDGQR(Q)	70	3.35E+05	1664.839
	(K)VQDLLLLDVAPLSLGIETAGGVMTALIPR(N)	66.2	1.13E+05	2975.685
	(K)NAVVTVPAYFNDAQR(Q)	67.7	8.90E+04	1664.839
	(K)EQTFSTYSDNQPGVLIQVYEGER(K)	68.7	1.46E+05	2660.247
	(R)LIGDSAK(N)	81.7	7.32E+04	703.399
beta-tubulin	Sequence	SPI (%)	Spectrum intensity	MH+ Matched (Da)
	(R)SGPYGQIFRPDNFVFGQTGAGNNWAK(G)	85.9	2.35E+05	2828.354
	(R)VSEQFTAMFR(R)	89.6	1.28E+05	1215.583
	(K)LAVNLVPFPR(L)	92.9	3.31E+05	1125.678
	(K)NSSYFVEWIPNNVK(A)	87	1.53E+05	1696.833
	(R)MMVTFSVVPSPK(V)	80.3	1.61E+05	1322.685
	(R)LHFFMIGFTPLTSR(G)	94	2.32E+05	1666.877
	(R)AILMDLEPGTMDSVR	88.2	3.13E+05	1647.808

(S)			
(K)EVDEQMLNVQNK(N)	78.6	1.15E+05	1446.689
(K)GHYTEGAELIDSVLDVVR(K)	88.3	2.74E+05	1972.997
(R)INVYFNEATGGR(Y)	82.2	1.78E+05	1340.659
(R)ALTVPELTQQMWDAK(N)	85.1	1.57E+05	1730.878
(R)YLTAAALFR(G)	84.9	2.78E+05	1025.578
(R)AILMDLEPGTMDSVR(S)	74.9	9.24E+04	1647.808
(R)EIVHIQGGQCGNQIGAK(F)	77.9	2.13E+05	1808.907
(R)SGPYGQIFRPDNFVFGQTGAGNNWAK(G)	80.3	2.09E+05	2828.354
(R)EIVHIQGGQCGNQIGAK(F)	92.6	1.99E+05	1808.907
(R)SGPYGQIFRPDNFVFGQTGAGNNWAK(G)	66.8	2.35E+05	2828.354
(K)MSATFVGNSTAVQEMFK(R)	74.4	5.65E+04	1847.867
(R)LHFFMIGFTPLTSR(G)	70.3	1.35E+05	1666.877

	(K)GHYTEGAELIDSVLDVVR(K)	77.2	1.65E+05	1972.997
	(K)NSSYFVEWIPNNVK(A)	68.7	7.39E+04	1696.833
	(K)NMMCAADPR(H)	68.3	7.22E+04	1065.427
	(K)GHYTEGAELIDSVLDVVR(K)	64.3	1.22E+05	1972.997

elongation factor-1 alpha-like protein	Sequence	SPI (%)	Spectrum intensity	MH+ Matched (Da)
	(R)VEQGIVKPNDEVR(F)	92.5	8.23E+05	1482.791
	(K)AGYSPIGFVR(C)	90.7	6.54E+05	1066.568
	(R)LIFELGGLPERELEK(L)	93.6	6.47E+05	1742.969
	(R)IAFLDGNTAVMLGK(V)	89.9	9.27E+05	1449.777
	(R)VEQGIVKPNDEVR(F)	96.7	7.86E+04	1482.791
	(K)HVEDFAAQIQTLDIPGELK(A)	100	1.07E+06	2124.097
	(R)LIFELGGLPER(E)	97.1	9.10E+05	1243.705
	(R)VPISGIYK(I)	89.8	4.17E+05	876.519
	(K)HVEDFAAQIQTLDIPG	87.6	1.01E+05	2124.097

	ELK(A)			
	(R)GVTISCTTK(E)	90.4	2.55E+05	966.492
	(K)MDADTAGYK(E)	81.4	1.55E+05	971.414
	(R)LLNLLGVR(Q)	88.8	1.37E+06	897.588
	(R)VEQGIVKPNDEVR(F)	73	7.25E+04	1482.791
	(K)EFFTDNWHYTIIDAPGHR(D)	65.8	2.63E+05	2219.03
	(K)RVDSAGPGDNVGMNIK(G)	80.3	1.94E+05	1629.801
	(R)QLCVGVNK(M)	91.6	4.15E+05	917.487
	(R)YTEIKDEMK(H)	61.3	2.05E+05	1156.555
	(R)LIFELGGLPERELEK(L)	93.1	2.74E+05	1742.969
	(R)QGDVMIMK(S)	77.7	4.00E+05	921.453
	(K)IKGVGDVLAGR(V)	66.9	1.04E+05	1084.647
	(R)LIFELGGLPER(E)	66.6	4.31E+04	1243.705

glyceraldehyde-3-phosphate dehydrogenase	Sequence	SPI (%)	Spectrum intensity	MH+ Matched (Da)
	(R)VPTPTVSIVDLVVQTEK(K)	91.6	7.14E+05	1825.032
	(K)VVAWYDNEWGYSQ	93.5	1.30E+05	1772.803

R(V)			
(K)NILEITDKPLVSIDFK(G)	87.8	7.16E+05	1845.037
(K)AVALVCPEVK(G)	93.1	4.68E+05	1085.602
(R)VPTPTVSIVDLVVQTEKK(T)	86.9	5.48E+05	1953.127
(K)TFAEEVNAAFK(E)	85.4	5.00E+05	1226.605
(R)VPTPTVSIVDLVVQTEK(K)	97.2	5.48E+05	1825.032
(K)GTMTTTHSYTGDQR(L)	71.8	2.19E+05	1555.68
(K)NILEITDKPLVSIDFK(G)	79.6	6.56E+05	1845.037
(K)VAINGFGR(I)	85.7	2.53E+05	833.463
(K)VVITAPGK(G)	77.9	3.71E+05	784.493
(K)NILEITDKPLVSIDFK(G)	74.3	1.98E+05	1845.037
(K)LNGIALR(V)	81.2	1.97E+05	756.473
(R)AAALNIVPTTTGAAK(A)	87.9	9.00E+05	1398.795
(K)NILEITDKPLVSIDFK(G)	73.3	2.49E+05	1845.037

	(K)HEYPIVSNASCTTNC MAPFVK(V)	80.2	1.67E+05	2426.094
	(R)VPTPTVSIVDLVVQT EK(K)	100	3.01E+05	1825.032
	(K)VVAWYDNEWGYSQ R(V)	72.3	1.10E+05	1772.803
	(K)HEYPIVSNASCTTNC MAPFVK(V)	71.2	1.83E+05	2426.094
	(K)VVITAPGK(G)	62.1	4.60E+04	784.493
	(K)HEYPIVSNASCTTNC MAPFVK(V)	63.1	1.55E+05	2426.094
	(K)FGIVK(G)	80.7	6.81E+04	563.355

alpha tubulin	Sequence	SPI (%)	Spectrum intensity	MH+ Matched (Da)
	(R)LIAQVISSLTASLR(F)	92	2.04E+05	1471.884
	(K)TIGGGDDAFNTFFSE TGAGK(H)	85.5	1.12E+05	1991.898
	(R)AVFLDLEPTVIDEVR(T)	91.3	4.91E+05	1715.921
	(R)AVFLDLEPTVIDEVR(T)	86.8	1.68E+05	1715.921

(R)AIFLDLEPTVVDEVR(T)	85.2	4.38E+05	1715.921
(R)IHFMLSSYAPVISAEK(A)	88.4	5.37E+05	1792.93
(R)TIQFVDWCPTGFK(C)	85.1	2.37E+05	1598.767
(K)YMACCLMYR(G)	67.7	1.02E+05	1267.509
(K)EIVDLCLDR(I)	89.5	2.22E+05	1132.567
(K)DVNAAVATIK(T)	84.7	2.38E+05	1001.563
(R)SLDIERPTYTNLNR(L)	82.1	4.46E+05	1691.871
(R)EDLAALEK(D)	73.1	1.07E+05	888.467
(K)AYHEQLSVAEITNSAFEPASMMAK(C)	71.9	2.41E+05	2625.232
(R)FDGALNVDVTEFQTNLVPYPR(I)	91.8	1.29E+05	2395.193
(K)CGINYQPPTVVPGGDLAK(V)	66.8	1.25E+05	1885.948
(K)EIVDTAIDR(I)	71.5	6.61E+04	1031.537
(K)CGINYQPPTVVPGGDLAK(V)	85.7	1.19E+05	1885.948
(R)LIAQVISSLTASLR(F)	61.8	7.40E+04	1471.884
(K)AYHEQLSVAEITNSAFEPASMMAK(C)	62.7	2.48E+05	2625.232

RUBISCO small subunit	Sequence	SPI (%)	Spectrum intensity	MH+ Matched (Da)
	(K)LPMFGCTNPNEVLG EIDSCKR(A)	98.2	1.33E+06	2437.131
	(R)LVAFDNIAQCQTIGFL VNRPPR(S)	91.3	6.17E+05	2529.34
	(R)FGNSASCNYYDNR(Y)	83.6	1.69E+05	1567.623
	(K)MFETFSFLPPLSHGEI AR(Q)	88.2	1.58E+06	2079.037
	(R)LVAFDNIAQCQTIGFL VNRPPR(S)	84.3	2.88E+05	2529.34
	(R)SGDFQSPENR(S)	83.6	1.59E+05	1136.497
	(R)SGDFQSPENR(S)	89.7	1.77E+05	1136.497
	(R)SGDFQSPENR(S)	78.9	1.83E+05	1136.497
	(K)MFETFSFLPPLSHGEI AR(Q)	67.1	1.30E+05	2079.037
	(R)LVAFDNIAQCQTIGFL VNRPPR(S)	71.4	2.14E+05	2529.34
	(K)MFETFSFLPPLSHGEI AR(Q)	69.7	1.28E+05	2079.037

	(R)LVAFDNIAQCQTIGFLVNRPPR(S)	62	1.47E+05	2529.34
	(R)AFPESYIR(L)	62.9	7.68E+04	982.499
	(R)SGDFQSPENR(S)	68.8	8.28E+04	1136.497
	(R)SGDFQSPENR(S)	63	3.70E+04	1136.497

actin	Sequence	SPI (%)	Spectrum intensity	MH+ Matched (Da)
	(R)VAPEEHPILLTEAPMNPK(L)	81.3	2.57E+05	1986.036
	(K)TYELPDGQVITVGSER(F)	87.6	9.87E+04	1763.881
	(K)AGFAGDDAPR(S)	74.7	7.37E+04	976.448
	(R)GYSFTTSAER(E)	64.6	5.52E+04	1118.511
	(K)YPIEHGIVTDWDDMEK(I)	71.7	1.27E+05	1947.879
	(R)VAPEEHPVLLTEAPLNPK(A)	68.4	9.62E+04	1954.064
	(R)GILALK(Y)	85	3.58E+04	614.424
	(R)DLTEYMMR(I)	76.6	7.72E+04	1058.465

elongation factor-1 alpha-like protein	Sequence	SPI (%)	Spectrum intensity	MH+ Matched (Da)

	(R)MFVLDEADEMLSR(G)	91.4	1.74E+05	1555.713
	(R)GIYAYGFEKPSAIQQK(G)	77.2	1.17E+05	1799.933
	(R)VLITTDLLAR(G)	81.9	2.66E+05	1114.683
	(R)ELAQQIEK(V)	62.7	6.77E+04	958.52
	(R)VYDMLR(R)	66.7	7.70E+04	796.402

sedoheptulose-1,7-biphosphatase	Sequence	SPI (%)	Spectrum intensity	MH+ Matched (Da)
	(R)ILFEVAPLALLVEK(A)	97.8	4.75E+05	1554.95
	(R)ILFEVAPLALLVEK(A)	92.1	1.12E+05	1554.95
	(R)TVFCIALK(D)	94.2	2.12E+05	951.533
	(R)YTGGMVPDVFQIIVK(E)	85.9	2.14E+05	1666.887
	(K)LLFEALR(Y)	93	3.74E+05	861.519
	(R)DQAAAGMGIYGPR(T)	92.8	8.58E+04	1306.621
	(R)YTGGMVPDVFQIIVK(E)	60.7	1.84E+05	1666.887
	(R)YTGGMVPDVFQIIVK(E)	67.6	1.61E+05	1666.887

heat shock protein 60	Sequence	SPI (%)	Spectrum intensity	MH+ Matched (Da)
	(K)LETTELEALGTAR(K)	81.9	5.48E+04	1403.738
	(K)ISSLQALMPLLEK(T)	100	7.13E+04	1442.829
	(K)AAVDEGIVPGGGAALLHASR(S)	65.8	8.55E+04	1860.993
	(K)TLENELEVVEGMK(F)	63.9	3.86E+04	1490.741

centrin	Sequence	SPI (%)	Spectrum intensity	MH+ Matched (Da)
	(R)EAFDLFDTDGSGTIDAK(E)	81.2	6.32E+04	1801.813
	(R)LFDDDETGK(I)	80.6	5.43E+04	1039.458
	(K)MISDIDKDGSGTIDFEEFLQMMTAK(M)	78.4	1.73E+05	2822.293
	(K)MISDIDKDGSGTIDFEEFLQMMTAK(M)	66.7	1.78E+05	2822.293
	(R)ALGFEPK(K)	78.7	7.93E+04	761.419

Ribosomal protein S5	Sequence	SPI (%)	Spectrum intensity	MH+ Matched (Da)
	(K)TIAECLADELINAAK(Q)	80.4	6.72E+04	1631.831

	(R)VNQAIYLLTTGAR(E)	89.6	4.67E+04	1419.795
	(K)TIAECLADELINAAK(Q)	80.2	6.72E+04	1631.831
	(K)TIAECLADELINAAK(Q)	100	4.58E+04	1631.831
	(R)IGSAGVVR(R)	100	4.63E+04	758.452

ascorbate peroxidase	Sequence	SPI (%)	Spectrum intensity	MH+ Matched (Da)
	(R)FDPEILHGANAGLK(N)	81.3	4.35E+05	1481.775
	(K)NALILLEPIK(A)	82.4	3.36E+05	1123.709

pyruvate orthophosphate dikinase	Sequence	SPI (%)	Spectrum intensity	MH+ Matched (Da)
	(R)VATDMVGEGLVTK(E)	73.2	4.53E+04	1319.688
	(R)LASLHEANPMLGFR(G)	62.8	8.47E+04	1555.805
	(R)AGISAVFR(S)	69.2	5.26E+04	820.468

RUBISCO large subunit	Sequence	SPI (%)	Spectrum intensity	MH+ Matched (Da)

(R)NSGLLLHIHR(A)	75.1	1.35E+05	1159.67
